# Synthetic and Structural Study of *peri*-Substituted Phosphine-Arsines

**DOI:** 10.3390/molecules26237222

**Published:** 2021-11-28

**Authors:** Brian A. Chalmers, D. M. Upulani K. Somisara, Brian A. Surgenor, Kasun S. Athukorala Arachchige, J. Derek Woollins, Michael Bühl, Alexandra M. Z. Slawin, Petr Kilian

**Affiliations:** 1 EaStChem School of Chemistry, University of St Andrews, St Andrews KY16 9ST, UK; bac8@st-andrews.ac.uk (B.A.C.); dmuks@st-andrews.ac.uk (D.M.U.K.S.); jdw3@st-andrews.ac.uk (J.D.W.); mb105@st-andrews.ac.uk (M.B.); amzs@st-andrews.ac.uk (A.M.Z.S.); 2 Treatt Plc, Bury St Edmunds IP32 7FR, UK; brian.surgenor@treatt.com; 3 Centre for Microscopy and Microanalysis, The University of Queensland, St Lucia, QLD 4072, Australia; kasun.athukorala@uq.edu.au; 4 Department of Chemistry, Khalifa University, Abu Dhabi 127788, United Arab Emirates

**Keywords:** *peri*-substitution, arsenic, organophosphorus, pnictine, single crystal X-ray structures

## Abstract

A series of phosphorus-arsenic *peri*-substituted acenaphthene species have been isolated and fully characterised, including single crystal X-ray diffraction. Reactions of EBr_3_ (E = P, As) with *i*Pr_2_PAcenapLi (Acenap = acenaphthene-5,6-diyl) afforded the thermally stable *peri*-substitution supported donor–acceptor complexes, *i*Pr_2_PAcenapEBr_2_ **3** and **4**. Both complexes show a strong P→E dative interaction, as observed by X-ray crystallography and ^31^P NMR spectroscopy. DFT calculations indicated the unusual As∙∙∙As contact (3.50 Å) observed in the solid state structure of **4** results from dispersion forces rather than metallic interactions. Incorporation of the excess AsBr_3_ in the crystal structure of **3** promotes the formation of the ion separated species [*i*Pr_2_PAcenapAsBr]^+^Br^−^ **5**. A decomposition product **6** containing the rare [As_6_Br_8_]^2–^ heterocubane dianion was isolated and characterised crystallographically. The reaction between *i*Pr_2_PAcenapLi and EtAsI_2_ afforded tertiary arsine (BrAcenap)_2_AsEt **7**, which was subsequently lithiated and reacted with PhPCl_2_ and Ph_2_PCl to afford cyclic PhP(Acenap)_2_AsEt **8** and acyclic EtAs(AcenapPPh_2_)_2_ **9**.

## 1. Dedication

This paper is dedicated to Prof Alex Slawin on the occasion of her 60th birthday, a world class crystallographer, a caring and compassionate colleague and mentor.

## 2. Introduction

Phosphines (R_3_P) are known as prototypical neutral donors (Lewis bases) with the most common examples being their use as tunable ligands in coordination chemistry [1,2]. Bonding occurs through donation of the phosphorus lone pair into an acceptor orbital on the metal (σ-component), often combined with the acceptance of electron density from the metal orbitals to empty orbitals on the phosphine (π-component). Arsines are also used as ligands, although their use is somewhat limited by their greater toxicity.

Although possessing a lone pair of electrons, both phosphines and arsines can also act as electron pair acceptors (Lewis acids) when equipped with electron withdrawing substituents, such as halogens [3].

For this paper, dative species in which a phosphine acts as donor, and another phosphine or arsine acts as acceptor, are of interest. Holmes experimented with various pnictogen compounds to form pnictogen–pnictogen donor–acceptor complexes, and in all cases, decomposition occurred at ambient temperatures [4,5]. For example, the reaction between Me_3_P and PCl_3_ (both colourless liquids) afforded a white solid of the composition (Me_3_P)_2_∙PCl_3_ at temperatures below freezing, the solid however decomposed upon warming [5]. Sisler then investigated the decomposition pathways and discovered redox degradation occurred readily at ambient conditions (e.g., Et_3_P + MePCl_2_ → Et_3_PCl_2_ + (MeP)_5_) [6].

**Figure 1:**
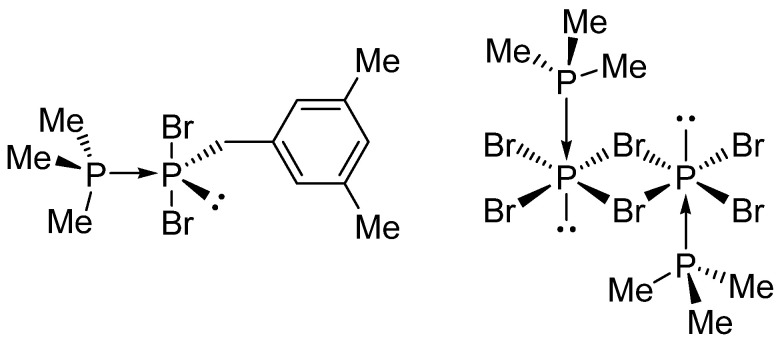
The first structurally characterised phosphine–phosphine donor–acceptor complexes.

It was not until 2001 that the first examples of phosphine–phosphine donor–acceptor species, Me_3_P→PBr_3_ and Me_3_P→Bz’PBr_2_ (Bz’ = 3,5-dimethylbenzyl), were structurally characterised by Müller and Winkler (Figure 1) [7]. Even so, these two adducts still decomposed at ambient conditions.

The first room temperature stable phosphine–phosphine donor–acceptor complex, (*i*Pr_2_PAcenapPCl_2_ (Acenap = acenaphthene-5,6-diyl), was isolated almost a decade later by us [8]. With *peri*-substitution enforcing the interaction between the two phosphorus atoms, the resulting strong dative interaction and reduced flexibility makes the redox decomposition pathways less accessible, allowing isolation and manipulation of these dative species at ambient temperatures.

Donor–acceptor species with heavier acceptor pnictogens were reported to be more stable owing to their reduced propensity to the redox decomposition pathways. Thus, while Me_3_P→PCl_3_ decomposes above −20 °C, [9] (Me_3_P)_2_→SbCl_3_ and Me_3_As→SbCl_3_ are stable at ambient conditions and melt with decomposition at 110 °C [10] and 145 °C [4,11], respectively. In his early study, Sisler reported on the prototypical R_3_Pn’→PnX_3_ pnictogen-pnictogen donor–acceptor complexes. Their stability was increased for heavier halogens in the acceptor species (I > Br > Cl > F) and followed the trend Sb > As > P for the Pn in the acceptor species (PnX_3_), while the stability trend As > P > Sb was observed for Pn’ in the donor species (R_3_Pn’) [9]. The stability of these and related dative species is likely to be driven by the difference in the Lewis acidity and basicity of the donor and acceptor (i.e., the strength of the dative bond), combined with the redox properties of the two components (i.e., the reducing and oxidising power of these). Comprehensive accounts on neutral [12] and related cationic species [13,14] have been published recently, encompassing the variety of the structural modes adopted by main group pnictine complexes.

**Figure 2:**
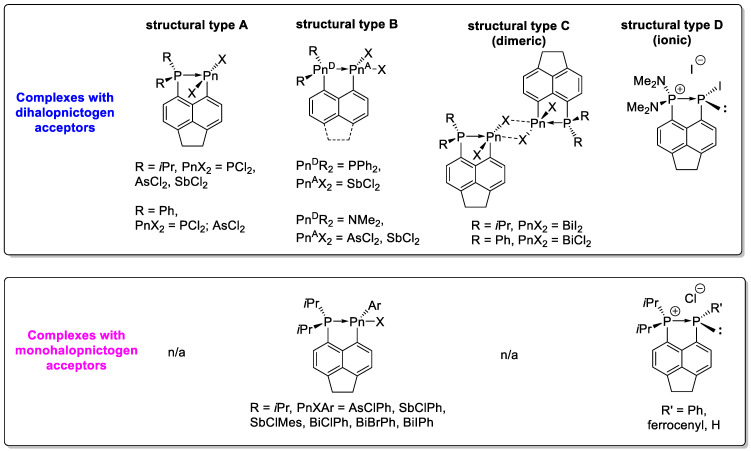
Selected structurally characterised *peri*-substituted pnictine-pnictine donor–acceptor complexes, indicating their structural diversity [8,15,16,17,18,19,20,21,22,23].

In the last two decades, we and others focused our attention on the *peri*-substituted pnictogen–pnictogen donor–acceptor complexes as shown in Figure 2.

Among complexes with dihalopnictines as acceptors, four structural types have been observed, including two molecular modes (**A**, **B**), µ-dichloro-bridged dimer **C** and fully ionic species **D**. Monohalopnictogen adducts attain two structural types, **D** (ionic) and **B** (molecular).

Motivated by the utility of dihalopnictines as precursor molecules in the development of new radical C–H coupling reactions [21], our attention turned to related species with dibromopnictine acceptor groups. Replacement of chlorine atoms with bromine was expected to alter thermal stability (through altering redox properties) and solubility in organic solvents (through altering the aggregation properties); both thermal stability and solubility are important for the synthetic utility of these dihalides. We also report on the chemistry of a geminal bis(acenaphthene)arsine, specifically its ability to tolerate treatment with *n*BuLi without cleavage of the As–C bonds. This opens up new synthetic pathways to further P and As *peri*-substituted species, some of which may serve as the C–H coupling precursors as mentioned above.

## 3. Results & Discussion

### 3.1. Reactions Involving PBr_3_ and AsBr_3_

**Scheme 1:**
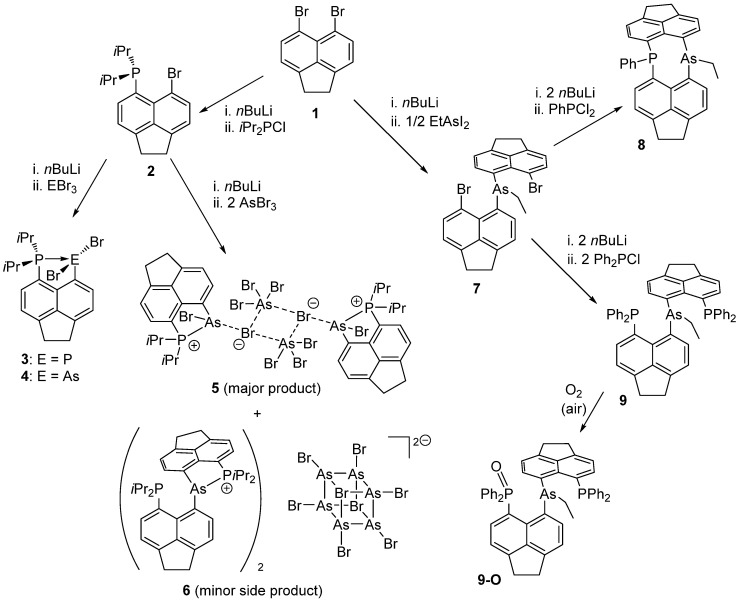
Syntheses reported in this paper.

In the syntheses described herein, 5,6-dibromoacenaphthene, **1**, was used as the principal precursor. Two subsequent lithium–halogen exchanges, followed by reactions with pnictogen halide electrophiles, were used to access the variety of *peri*-substitution patterns shown in Scheme 1.

5-Bromo-6-(diisopropylphosphino)acenaphthene **2** was prepared using our literature procedure [8]. Lithiation of **2** using *n*BuLi, followed by a slow addition of the formed *i*Pr_2_PAcenapLi to a fivefold excess PBr_3_, gave the phosphonium-phosphoranide **3** (*i*Pr_2_PAcenapPBr_2_) in a good yield (65%). The related phosphonium-arsoranide **4** (*i*Pr_2_PAcenapAsBr_2_) was made using the same procedure in ~75% yield; however, in this case, only one molar equivalent of AsBr_3_ was used in the reaction. The selectivity of the reactions of aryllithiums towards pnictogen halides PnX_3_ (Pn = pnictogen) is affected greatly by the order and rate of the reactant addition and the stoichiometric ratio of the reagents. A large excess of the pnictogen halide is often necessary in order to promote monosubstitution giving R_2_PAcenapPnX_2_, i.e., to avoid geminal di- or trisubstitution, leading to the formation of (R_2_PAcenap)_2_PnX or (R_2_PAcenap)_3_Pn [8,15,17,22,24,25]. In the preparation of **3**, the excess PBr_3_ was necessary to achieve good yields; the excess PBr_3_ was removed by careful washing with THF and diethyl ether.

Compounds **3** and **4** represent an expansion of the small number of phosphine-phosphine and phosphine–arsine donor–acceptor complexes, that are stable (isolable) at ambient temperatures. The unsupported phosphine–phosphine complexes undergo redox degradation at temperatures above –40 °C or at even lower temperatures [7]. Both **3** and **4** can be stored indefinitely under inert atmosphere at ambient temperature in the solid form, but **3** undergoes decomposition slowly (over the course of a week) in common organic solvents (thf, dcm, chloroform). The arsenic congener **4** immediately decomposes in chlorinated solvents, giving a mixture of insoluble products. Both **3** and **4** are air sensitive. It should be noted that the thermal stability and air sensitivity of **3** and **4** is rather similar to that of the chlorine congeners *i*Pr_2_PAcenapECl_2_ (E = P, As) [8,15].

The ^31^P{^1^H} NMR spectrum of **3** consists of two doublets at δ_P_ 65.3 (*i*Pr_2_P) and 32.9 ppm (PBr_2_) with a large ^1^*J*_PP_ magnitude of 353.7 Hz (AX spin system). This is in good agreement with the relevant data of *i*Pr_2_PAcenapPCl_2_ (δ_P_ 68.8 and 40.4 ppm, ^1^*J*_PP_ 363 Hz) [8]. The ^31^P{^1^H} NMR spectrum of **4** (singlet at δ_P_ 56.6 ppm) is in agreement with the previously published data for *i*Pr_2_PAcenapAsCl_2_ (δ_P_ 65.3 ppm) [15]. This strongly suggests the structures of **3** and **4** in the solution are similar to those of their chlorine congeners. Significant deshielding of the donor (*i*Pr_2_P) phosphine group in **3** and **4** compared to that in **2** (δ_P_ –2.2 ppm) strongly suggests major sequestration of the lone pair electron density takes place by the pnictogen halide (PnX_2_) acceptor group. Compounds **3** and **4** were fully characterised including ^1^H, ^13^C{^1^H} and ^31^P{^1^H} NMR, IR and MS.

**Figure 3:**
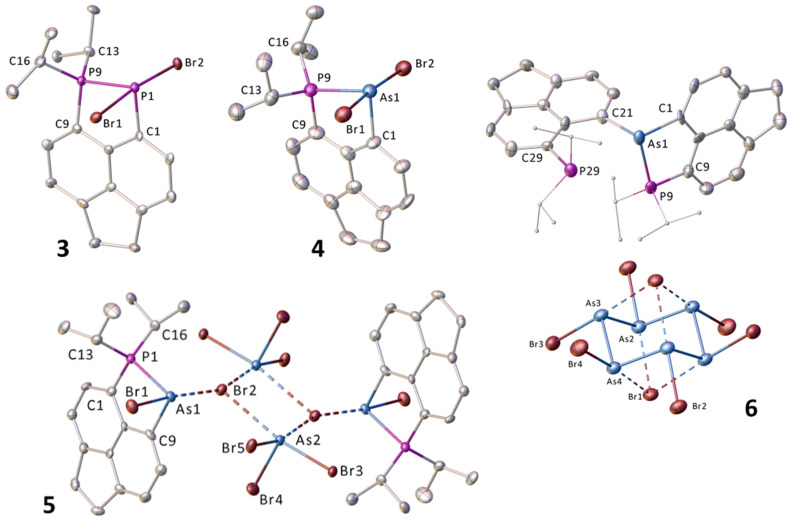
Crystal structures of **3**–**6**. Hydrogen atoms and solvating molecules (THF in **3** and benzene in **4**) are omitted. The second molecule in the asymmetric unit of **4** is omitted. **5** forms centrosymmetric dimer, two asymmetric units are shown in the figure. Monocation of **6** is shown at the top, the [As_6_Br_8_]^2−^ dianion is at the bottom. Thermal ellipsoid ds are plotted at the 40% level.

The crystal structures of **3** and **4** are shown in Figure 3 and Table 1. The diffraction data indicate a strong dative interaction is formed between the two phosphorus atoms in **3**, with a P–P bond length of 2.2701(16) Å, which is just within a range of standard P–P single bond (2.22 ± 0.05 Å). Two molecules of **4** are present in the asymmetric unit. The P–As distances in these (2.405(3) and 2.407(3) Å) are crystallographically identical, consistent with a strong dative bond, and comparable to a standard P−As single bond length. Based on the electronegativity considerations, the ECl_2_ group is expected to be better acceptor than the respective EBr_2_ group (E = P, As). However, no dramatic lengthening of the P→EBr_2_ vs. the P→ECl_2_ dative bond is observed; the P−As bond length in the chlorine congener *i*Pr_2_P-Acenap-AsCl_2_ (2.4029(7) Å) [15] is crystallographically identical to that in **4**, while the P−P bond length in *i*Pr_2_PAcenapPCl_2_ (2.2570(14) Å) [8] is only marginally shorter than that in **3.**

Although neither **3** nor **4** form a halogen bridged dimeric assemblies in the crystal, the P–Br bond lengths are nonidentical within **3** (P1–Br1 2.6864(13) Å; P1–Br2 2.4376(13) Å). In contrast, the As–Br bond lengths are almost identical in **4**: As1–Br1 2.6337(19) [2.6339(19)] Å, As1–Br2: 2.6400(18) [2.664(2)] Å (values in square brackets are for the second molecule in the asymmetric unit). The P–Br and As–Br bond lengths in **3** and **4** indicate an intermediate character between ionic and covalent bonds; their elongation is rather significant when compared to the bond lengths in the covalent PBr_5_ (P–Br: 2.221 and 2.158 Å) [26] and AsBr_3_ (in adduct with hexaethylbenzene, P–As 2.322(1) Å) [27]. The acceptor pnictogen atoms in **3** (P1) and **4** (As1) adopt a pseudo-trigonal bipyramidal geometry, with the two bromine atoms occupying the axial positions (Br−E−Br 172.22(5)° in **3** and 173.95(6)° [172.27(6)°] in **4**). The P9 and C1 atoms occupy the equatorial positions, with the 3rd position taken up by the lone pair. The inequality and elongation of the phosphorus–halogen bonds are phenomena observed in all relevant structurally characterised phosphine–phosphine and phosphine–arsine donor–acceptor complexes, and presumably stem mainly from packing effects [7,8,17]. The equal length of As–Br bonds, observed within **4**, is likely a result of the symmetrical nature of the local polarity effects around the bromine atoms within the crystal structure of **4**. The acenaphthene framework in both **3** and **4** encounters only minimal in-plane and out-of-plane distortions from the ideal (planar) geometry, consistent with a relaxed *peri*-region (P–Pn bonded) structures.

**Figure 4:**
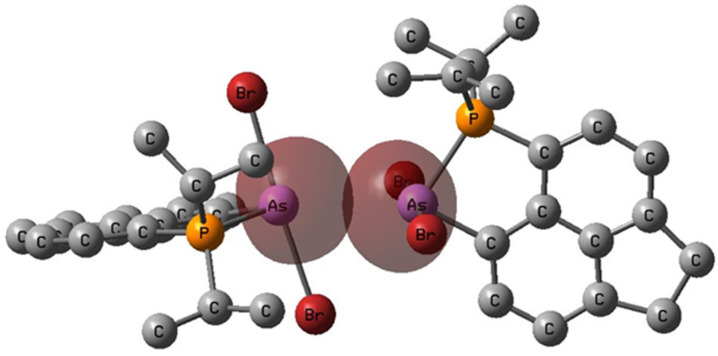
Plot demonstrating overlap of the lone pairs on arsenic atoms in a pair of molecules **4**. Natural bond orbitals, B3LYP/6-31(+)G* level on B3LYP-D3/6-31(+)G* optimised geometry, isovalue for plot 0.04 a.u.; hydrogen atoms omitted for clarity.

An interesting feature was observed in the crystal packing of **4**. Rather than forming a Br···As intermolecular close contacts, commonly seen in halogen-bridged dimers [7,16], pairs of molecules of **4** are oriented so that a short (intermolecular) As···As contact (3.50 Å) is formed. To gain some insight into significance of this, we performed density functional theory (DFT) computations on a ‘dimer’ motif, carved out of the crystal. Because the interaction between the two molecules was expected to be weak, a level was chosen that contains corrections for attractive van der Waals forces (dispersion) and basis-set superposition error (BSSE; an intrinsic error for intermolecular interaction energies with finite basis sets), denoted B3LYP-D3/6-31(+)G* (see SI for details; B3LYP functional and basis sets are the same as in our previous studies on *peri*-acenaphthene derivatives) [16,21]. Interestingly, full geometry optimisation of the dimer at that level affords an even shorter As···As contact (3.28 Å) than that observed in the solid, and a substantial binding energy (ΔE = –15.7 kcal mol^−1^, estimated ΔH^298^ –14.4 kcal mol^−1^ and ΔG^298^ –3.43 kcal mol^−1^). Despite this sizeable interaction energy, only small covalent contributions to the binding are apparent from the computed intermolecular Wiberg bond indices (WBIs) [28], which do not exceed 0.05 for both As···As and As···Br contacts. Closer inspection of the contributions to the binding energy ΔE reveals that it is entirely dominated by dispersion interactions: the -D3 contribution in the optimised structure amounts to –16.5 kcal mol^−1^, i.e., the interaction would be expected to be repulsive at the B3LYP level without explicit dispersion correction. Indeed, if the structure is relaxed at the B3LYP/6-31(+)G* level (still BSSE-corrected), the dimer essentially falls apart. In this scenario, a much longer As···As distance ensues (3.87 Å) with a very weak binding energy ΔE = –2.1 kcal mol^−1^, presumably due to weak electrostatic interactions. It is remarkable that the pairs of molecules are held together by such strong dispersion forces, as it brings the arsenic atoms (with their lone pairs pointing toward each other) to an essentially repulsive distance, as shown in Figure 4.

Conducting the reaction of *i*Pr_2_PAcenapLi with 2 molar equivalents of AsBr_3_ gave **5** as a pale-yellow solid, which was contaminated with residual LiBr. Crystals suitable for diffraction work were grown from acetonitrile. The crystal structure of **5** is shown in Figure 3 and Table 1. Compound **5** is formed by an ion pair [*i*Pr_2_PAcenapAsBr]^+^Br^−^, co-crystallised with an AsBr_3_ molecule (see Scheme 1), with two acenaphthene units and PBr_3_ molecules forming a centrosymmetric assembly. All Br∙∙∙As distances around the μ^3^-bridging Br2 atom (3.00–3.04 Å) are significantly elongated compared to all other As–Br bonds (2.3370(9)–2.4375(9) Å), supporting the interpretation of the bonding as ionic. Comparing the structures of **4** and **5** shows no significant change in the P–As bond length (2.3978(15) Å in **5**). The most substantial changes are the shortening of the As1–Br1 bond to 2.4375(9) Å and the elongation of the other distance, As1···Br2, to 3.000(9) Å (c.f. As–Br bond lengths 2.634(2)–2.6639(19) Å in **4**).

In one incidence, while attempting to grow crystals of **5** from boiling acetonitrile, a small crop of colourless crystals was obtained. These were subjected to single-crystal X-ray diffraction and were found to be compound **6**, consisting of a complex phosphonium cation and an unusual octabromohexaarsenate ([As_6_Br_8_]^2–^) dianion (Scheme 1, Figure 3 and Table 1). The cation of **6** consists of two acenaphthene groups geminally attached to an arsenic atom. The two P–As distances are very disparate, indicating the presence of a standard P9–As1 bond (2.365(4) Å), and an onset of 3-center 4-electron P···As–C interaction (P29···As1 3.145(4) Å; P29···As1–C1 angle is 176.9(3)°). A tentative mechanism of the cation formation involves an attack of the second molecule of *i*Pr_2_PAcenapLi on **4** or **5**. Only one previous incidence of crystallographic characterisation of octabromohexaarsenate dianion was found in the literature; this was in the form of its tetraphenylphosphonium salt [29]. The dianion of **6** is essentially isostructural to the previously reported example, both can be seen as an (AsBr)_6_ oligoarsine ring in a chair conformation, capped by two bromide anions to form a heterocubane cluster. A tentative mechanism of formation of **6** involves thermally induced reduction of AsBr_3_ (present in the structure of **5**), leading to the formation of cyclohexaarsine (AsBr)_6_. This is followed by coordination by bromide anions to give the [As_6_Br_8_]^2–^ dianion. The reduction step may be analogous to that seen throughout the chemistry of pnictine–pnictine complexes, first described by Sisler [6], in which the trihalide halogenates the tertiary phosphine moiety (to halophosphonium [R_3_P^V^Cl]^+^Cl^-^), reducing itself to (As^I^X)_n_. This proposed mechanism is distinct (but related) to that proposed by Macdonald for formation of octaiodocyclohexaarsenates, where the diphosphine ligand (L) cleavage from pre-assembled arsenic(I) species [LAs][I] was suggested [30]. Further characterisation of **6** was not possible due to the small amount of crystals available. Attempts to repeat synthesis of **6** on a larger scale were not successful.

Our attempts to prepare the iodine congener *i*Pr_2_PAcenapPl_2_ via reaction of *i*Pr_2_PAcenapLi with PI_3_ or P_2_I_4_ gave complex mixtures as judged by ^31^P NMR. On a similar note, reactions of *i*Pr_2_PAcenapLi with SbBr_3_ gave inseparable mixtures, presumably via undesirable redistribution and redox processes [9]. This is in stark contrast to the analogous reaction of *i*Pr_2_PAcenapLi with SbCl_3_, which afforded the desired donor–acceptor complex *i*Pr_2_PAcenapSbCl_2_ in an excellent yield [16].

### 3.2. Reactions Involving EtAsI_2_

To expand the range of arsenic *peri*-substituted species, reactions with ethyldiiodoarsine have been studied. Monolithiation of 5,6-dibromoacenaphthene, followed by an addition of the formed *i*Pr_2_PAcenapLi to one molar equivalent of EtAsI_2_, was expected to yield iodoarsine Et(I)AsAcenapBr. However, the ^1^H NMR spectrum of the white powder obtained after workup indicated a product of a double (geminal) substitution, a tertiary arsine **7**, has been formed solely (Scheme 1). The reaction was repeated with highly diluted EtAsI_2_ and very slow addition rate to limit the double substitution, however even then, **7** was the sole product of the reaction.

**Figure 5:**
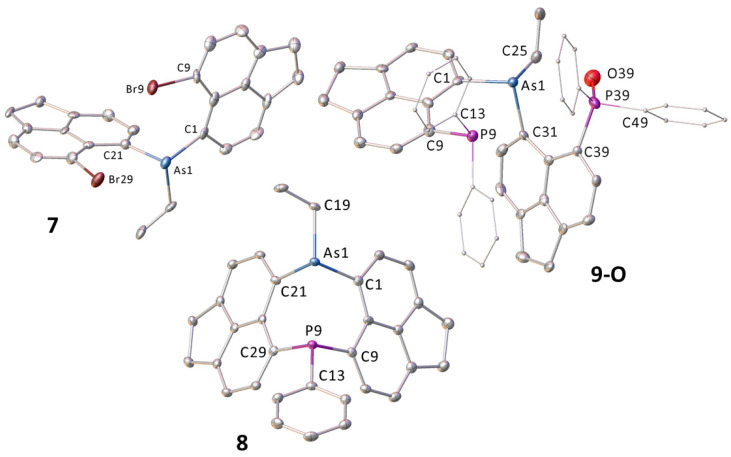
Crystal structures of **7**, **8** and **9-O**. Hydrogen atoms and solvating molecules (CH_2_Cl_2_ in **7** and **8**) are omitted. Thermal ellipsoids are plotted at the 40% level.

The crystal structure of **7** is shown in Figure 5 and Table 1. The arsenic atom adopts trigonal pyramidal geometry. The As∙∙∙Br distances (3.23(1) and 3.28(1) Å, 82–83% of the sum of the respective Van der Waals radii [31]) as well as the large splay angles (17(1) and 19(1)°) indicate repulsive interactions in the *peri*-region, with As∙∙∙Br interactions forced through *peri*-geometry.

Following the clean synthesis of **7**, we were interested in its use as a synthon, particularly its ability to undergo lithium–halogen exchange, which would open many options for its further functionalisation. Initially, we focussed on introducing phosphorus moieties to the *peri*-positions. Synthesis of two examples, cyclic (**8**) and acyclic (**9**), resulted from these efforts (Scheme 1). Using standard low-temperature lithium–halogen exchange conditions, double lithiation of **7** was incomplete after several hours at −78 °C, despite using a slight excess over two equivalents of *n*-butyllithium. To promote the double lithiation we have adopted conditions similar to those reported by Kasai for double lithiation of 5,6-dibromoacenaphthene [32], which included the addition of chelating base tetramethyleethylenediamine, TMEDA. In addition, we have switched to a more polar reaction solvent (thf). These changes sufficiently stabilised the dilithiated species EtAs(AcenapLi)_2_, as the subsequent reaction with PhPCl_2_ gave cyclic species **8** in a good yield (ca. 70%). Unfortunately, the workup did not allow for complete removal of the salt impurities as indicated by the results of the elemental analysis, meaning the yield mentioned above is only approximate. **8** showed a sharp singlet at δ_P_ –26.5 ppm and was only barely soluble in common organic solvents, which precluded acquisition of good quality ^13^C{^1^H} NMR spectra.

Reacting the in situ formed dilithiated species EtAs(AcenapLi)_2_ with two molar equivalents of chlorodiphenylphosphine in thf afforded **9** as the sole phosphorus-containing product as confirmed by ^31^P{^1^H} NMR spectroscopy (singlet at δ_P_ −19.9 ppm). **9** was found to be insoluble in many common organic solvents and only sparingly soluble in dichloromethane. In an attempt to grow crystals of **9** suitable for X-ray work, colourless needle shaped crystals were obtained by diffusion of diethyl ether into a saturated solution of **9** in dichloromethane. The X-ray diffraction showed that partial oxidation (presumably by air) has taken place in this sample, giving the phosphine oxide species **9-O**. Notably, the presence of the oxidised species **9-O** was not evident in the bulk of the reaction product **9** by any other characterisation results such as elemental analysis and mass spectroscopy. Compounds **7**–**9** were fully characterised, including ^1^H, ^13^C{^1^H} NMR (where solubility allowed this), IR, Raman and MS.

The crystal structures of **8** and **9-O** are shown in Figure 5 and Table 1. The central part of the molecule of **9** adopts the shape of a puckered (8-membered) ring, with the angle between the two acenaphthene planes being 89°. The molecule of **9-O** is rather crowded with large groups attached to the two *peri*-regions. As mentioned, one of the phosphorus environments in **9-O** is partially oxidised (50% occupancy) to the phosphine oxide (from accidental exposure to air).

The P∙∙∙As distance comparison in **8** and **9-O** is rather interesting. The As1∙∙∙P9 distance in **9-O** is 3.176(5) Å, while the relevant distance of 3.004(6) was observed in **8**. This indicates the buttressing effect of the double *peri*-strain in **8** is rather significant, shortening the P∙∙∙As distance by 0.17 Å, i.e., by 4.2% of the sum of the respective Van der Waals radii [31]. The large positive splay angles indicate repulsive interactions in the *peri*-regions of **7**, **8** and **9-O**, with the in-plane distortions being the major mechanism of strain relaxation, although in **9-O** the arsine group (As1) shows also large out-of-plane displacement (0.71 Å) from one of the acenaphthene mean planes.

## 4. Experimental

### 4.1. General Considerations

**Figure 6:**
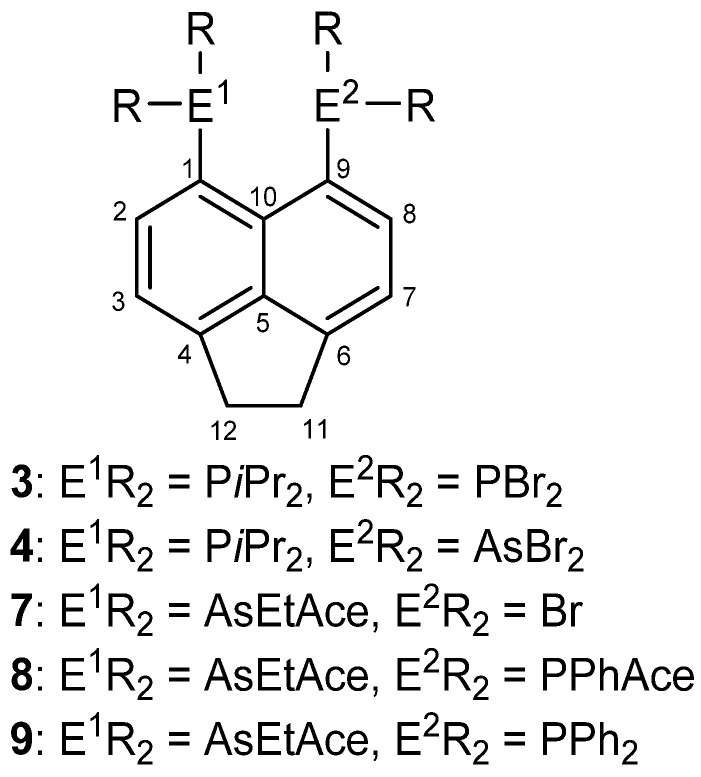
General NMR numbering scheme.

All reactions and manipulations were carried out under an atmosphere of nitrogen using standard Schlenk techniques or under an argon atmosphere in a Saffron glove box. Dry solvents were either collected from an MBraun Solvent Purification System, or dried and stored according to common procedures [33]. Compounds **1** and **2** were prepared according to literature procedures [8,34]. Arsenic tribromide was prepared using a modified version of the published procedure (see Supplementary Materials) [35]. EtAsI_2_ was prepared as described in the literature [36]. Arsenic oxide (>99.9%) was purchased from Alfa Aesar and used as received. Other chemicals were purchased from commercial sources and used as received. Further experimental details are provided in SI. NMR numbering scheme is shown in Figure 6.

Caution! Arsenic halides are highly toxic powerful vesicants, which cause severe irritation and blistering if allowed to come in contact with skin. Suitable precautions, including the use of neoprene or rubber gloves, should be taken when handling these.

### 4.2. Synthetic Methods

#### 4.2.1. iPr_2_PAcenapPBr_2_ (**3**)

To a cooled (–78 °C) rapidly stirring solution of **2** (2.60 g, 7.4 mmol) in diethyl ether (60 mL), *n-*butyllithium (3.0 mL, 2.5 M in hexane, 7.5 mmol) was added dropwise over 1 h and the mixture was left to stir for 2 h at the same temperature. The resulting suspension of *i*Pr_2_PAcenapLi was added via cannula (in small batches) over 1 h to a rapidly stirring solution of phosphorus tribromide (9.90 g, 3.5 mL, 36.7 mmol) in diethyl ether (30 mL), cooled to –78 °C. The reaction mixture was left to stir and warm to ambient temperature overnight. The orange suspension was filtered and the solid collected was washed with THF (30 mL) followed by diethyl ether (30 mL) to remove excess PBr_3_ and partially also LiBr. After drying in vacuo, **3** (contaminated with LiBr) was obtained as a yellow powder (2.20 g, ~65%). Note that it is important when filtering the compound not to let the solid settle and compact as this will prevent removal of all PBr_3_. Crystals of **3** suitable for X-ray diffraction were grown from thf. Small scale recrystallisation from chloroform afforded analytically pure material. M. p. 168–172 °C. **Elemental Analysis** Calcd. (%) for C_18_H_22_P_2_Br_2_: C 46.99, H 4.82; Found: C 47.07, H 4.87. **^1^H NMR**: δ_H_ (400.1 MHz, CDCl_3_) 8.82 (1H, dd (~t), ^3^*J*_HH_ = 6.8, ^3^*J*_HP_ = 1.2 Hz, H-8), 8.17 (1H, dd, ^3^*J*_HH_ = 7.2, ^3^*J*_HP_ = 5.1 Hz, H-2), 7.73 (1H, dd, ^3^*J*_HH_ = 6.8, ^4^*J*_HP_ = 2.4 Hz, H-7), 7.62 (1H, dd, ^3^*J*_HH_ = 7.2, ^4^*J*_HP_ = 2.8 Hz, H-3), 4.29–4.14 (2H, m (~septet), ^3^*J*_HH_ = 7.0 Hz, *CH*(CH_3_)_2_), 3.58 (4H, br s, H-11 and H-12), 1.57 (6H, ^2^*J*_HP_ = 19.2, ^3^*J*_HH_ = 7.0 Hz, 2 × CH_3_), 1.50 (6H, dd, ^2^*J*_HP_ = 19.5, ^3^*J*_HH_ = 7.0 Hz, 2 × CH_3_). **^13^C{^1^H} NMR**: δ_C_ (100.6 MHz, CDCl_3_) 154.4 (s, qC-6), 151.8 (s, qC-4), 140.3 (dd, ^2^*J*_CP_ = 20.5, ^2^*J*_CP_ = 3.4 Hz, qC-10), 139.0 (dd, ^3^*J*_CP_ = 11.0, ^3^*J*_CP_ = 1.5 Hz, qC-5), 137.5 (br s, C-8), 134.8 (dd, ^2^*J*_CP_ = 31.5, ^3^*J*_CP_ = 8.5 Hz, C-2), 129.8 (dd, ^1^*J*_CP_ = 46.4, ^2^*J*_CP_ = 4.7 Hz, qC-1), 122.6 (s, C-3), 122.5 (s, C-7), 112.8 (dd, ^1^*J*_CP_ = 55.7, ^2^*J*_CP_ = 8.0 Hz, qC-9), 31.6 (s, C-11 or C-12), 31.2 (s, C-11 or C-12), 27.5 (dd, ^1^*J*_CP_ = 28.5, ^2^*J*_CP_ = 4.5 Hz, *CH*(CH_3_)_2_), 18.8 (br s, 2 × CH_3_), 18.3 (br s, 2 × CH_3_). **^31^P{^1^H} NMR**: δ_P_ (162.0 MHz, CDCl_3_) 65.3 (d, P_PBr2_), 32.9 (d, P_PiPr2_), ^1^*J*_PP_ = 353.7 Hz. **IR** (KBr disc, cm^–1^) ν = 2963 vs, 2934 vs, 2862 s, 1457 s, 1443 s, 839 m, 640 s. **HRMS (APCI+)**: *m/z* (%); Calcd. for C_18_H_22_P_2_Br (M–Br): 379.0375, found 379.0376 (100).

#### 4.2.2. iPr_2_PAcenapAsBr_2_ (**4**)

Compound **4** was prepared using the same procedure as per compound **3** except using the following: **2** (2.00 g, 5.7 mmol) in diethyl ether (40 mL), *n*–butyllithium (2.3 mL, 2.5 M in hexanes, 5.7 mmol) and arsenic tribromide (1.80 g, 5.7 mmol) in diethyl ether (50 mL). Addition time of *i*Pr_2_PAcenapLi to AsBr_3_ was 3 h. **4** was isolated as a yellow powder (2.17 g, ~75%). M. p. 159–161 °C. Crystals suitable for X-ray diffraction work were grown from benzene at room temperature. Contamination with residual LiBr prevented satisfactory microanalysis and accurate determination of the yield. **^1^H NMR**: δ_H_ (500.1 MHz, C_6_D_6_) 7.92 (1H, d, ^3^*J*_HH_ = 7.1 Hz, H-8), 7.37 (1H, dd, ^3^*J*_HP_ = 8.6, ^3^*J*_HH_ = 7.2 Hz, H-2), 7.01 (1H, dt, ^3^*J*_HH_ = 7.2, ^4^*J*_HH_ = 1.5 Hz, H-7), 6.99 (1H, dt, ^3^*J*_HH_ = 7.2, ^4^*J*_HH_ = 1.4 Hz, H-3), 3.61 (2H, septet, ^3^*J*_HH_ = 7.2 Hz, *CH*(CH_3_)_2_), 2.89–2.73 (4H, m, H-11 and H-12), 1.36 (6H, dd, ^3^*J*_HP_ = 18.9, ^3^*J*_HH_ = 7.2 Hz, 2 × CH_3_), 1.14 (6H, dd, ^3^*J*_HH_ = 16.6, ^3^*J*_HH_ = 7.3 Hz, 2 × CH_3_). **^13^C{^1^H} NMR**: δ_C_ (125.8 MHz, C_6_D_6_) 153.2 (s, qC-4), 146.5 (s, qC-9), 145.8 (s, qC-5), 140.7 (d, ^1^*J*_CP_ = 22.3 Hz, qC-1), 134.1 (s, C-2), 131.2 (d, ^3^*J*_CP_ = 7.5 Hz, C-8), 122.2 (s, C-7), 119.5 (d, ^3^*J*_CP_ = 9.5 Hz, C-3), 30.8 (s, C-11 or C-12), 30.2 (s, C-11 or C-12), 27.6 (d, ^1^*J*_CP_ = 26.3 Hz, *CH*(CH_3_)_2_), 20.0 (s, 2 × CH_3_), 17.7 (d, ^2^*J*_CP_ = 5.7 Hz, 2 × CH_3_). **^31^P{^1^H} NMR**: δ_P_ (202.5 MHz, C_6_D_6_) 56.6 (s). **MS** (EI): *m/z* (%) 422.97 (20) [M–Br], 344.04 (95) [M–2Br], 300.99 (90) [*i*PrPAcenapAs], 257.95 (95) [AcenapPAs], 234.74 (100) [AsBr_2_].

#### 4.2.3. [iPr_2_PAcenapAsBr]^+^Br^–^∙AsBr_3_ (**5**) and [(iPr_2_PAcenap)_2_As]_2_[As_6_Br_8_] (**6**)

To a cooled (–78 °C) rapidly stirring solution of **2** (2.00 g, 5.7 mmol) in diethyl ether (40 mL), *n-*butyllithium (2.3 mL, 2.5 M in hexane, 5.7 mmol) was added dropwise over 1 h and the mixture was left to stir for 2 h at the same temperature. The resulting suspension of *i*Pr_2_PAcenapLi was added via cannula (in small batches) over 1 h to a rapidly stirring solution of arsenic tribromide (3.60 g, 11.5 mmol) in diethyl ether (80 mL), cooled to –78 °C. The reaction mixture was left to stir and warm to ambient temperature overnight. The suspension was filtered and the solid collected was washed with diethyl ether (30 mL). After drying in vacuo, **5** (contaminated with LiBr) was obtained as a pale-yellow powder (3.84 g, ca. 81%). Crystals of **5** suitable for X-ray diffraction work were grown from THF at room temperature. Contamination with LiBr prevented satisfactory microanalysis and accurate determination of the yield. Solution NMR spectra of **5** are identical to those of **4**.

Few crystals of **6** suitable for diffraction work were obtained from recrystallisation of the crude product **5** from hot acetonitrile. No further characterisation was possible due to small amount available.

#### 4.2.4. (BrAcenap)_2_AsEt (**7**)

A solution of *n*-butyllithium (12.8 mL of 2.5 M solution in hexanes, 32 mmol) was added dropwise to a rapidly stirred suspension of 5,6-dibromoacenaphthene **1** (10.0 g, 32 mmol) in THF (120 mL) at −78 °C. The mixture was maintained for 2 h at this temperature. To this, a solution of ethyldiiodoarsine (1.95 mL, 16 mmol) in THF (20 mL) was added dropwise over one hour at −78 °C. The resulting suspension was left to warm to room temperature overnight. The volatiles were removed in vacuo. Dichloromethane (50 mL) was added, and the resulting suspension was filtered. The product was obtained as a white powder after removal of the volatiles from the filtrate in vacuo. Recrystallisation of the crude material from dichloromethane gave **7** as colourless needle crystals (5.37 g, 82%), some of these were suitable for X-ray diffraction work. M. p. 199–200 °C. **Elemental analysis**: Calcd. (%) for C_26_H_21_AsBr_2_: C 54.96, H 3.72; found: C 54.86, H 3.65. **^1^H NMR**: δ_H_ (400.1 MHz, CD_2_Cl_2_) 7.60 (2H, d, ^3^*J*_HH_ = 7.4 Hz, H-8), 7.48 (2H, d, ^3^*J*_HH_ = 7.2 Hz, H-2), 7.08 (2H, d, ^3^*J*_HH_ = 7.2 Hz, H-3), 7.00 (2H, d, ^3^*J*_HH_ = 7.4 Hz, H-7), 3.29–3.19 (8H, m, H-11 and H-12), 2.04 (2H, q, ^3^*J*_HH_ = 7.7 Hz, As-CH_2_), 1.19 (3H, t, ^3^*J*_HH_ = 7.6 Hz, CH_3_). **^13^C{^1^H} NMR**: δ_C_ (100.6 MHz, CD_2_Cl_2_) 146.2 (s, qC), 145.7 (s, qC), 140.7 (s, qC), 135.6 (s, qC), 134.6 (s, C-2), 133.0 (s, C-8), 132.3 (s, qC), 119.4 (s, C-3), 119.2 (s, C-7), 115.5 (s, qC), 29.1 (s, C-11 or C-12), 28.8 (s, C-11 or C-12), 23.0 (s, As-CH_2_), 10.4 (s, CH_3_). **IR** Data (KBr disc, cm^−1^): ν = 3025 w (ν_ArH_), 2920 s, 2863 w (ν_CH_), 1603 s, 1411 s, 1320 vs, 1251 m, 1200 m, 1100 m, 1044 m, 836 vs, 738 m, 602 m, 543 m. **Raman** (glass capillary, cm^−1^) ν = 3063 m (ν_ArH_), 2952 m, 2932 s, (ν_CH_), 1608 m, 1561 vs, 1442 vs, 1412 s, 1345 m, 1320 vs, 815 m, 702 s, 577 vs, 557 s, 290 vs. **MS (ES+)** *m/z* (%) 591 (100) [M + Na]. **HRMS**: *m/z* Calcd. for C_26_H_21_Br_2_AsNa (M + Na): 590.9103, found 590.9094.

#### 4.2.5. Cyclo-PhP(Acenap)_2_AsEt (**8**)

*N*,*N*,*N*′,*N*′–tetramethylethylene-1,2-diamine (TMEDA, 0.51 mL, 3.4 mmol) was added to a suspension of **7** (0.75 g, 1.32 mmol) in THF (35 mL) and the mixture was cooled to –78 °C. A solution of *n*-butyllithium (1.26 mL, 2.5 M in hexanes, 3.2 mmol) was added dropwise with stirring over 1 h. The solution was maintained at –78 °C for 30 min before being warmed up and maintained at 0 to 10 °C for an hour. The solution was re-cooled to –78 °C and a solution of PhPCl_2_ (0.18 mL, 1.32 mmol) in THF (5 mL) was added dropwise. The mixture was left to warm up to room temperature overnight with stirring. The volatiles were removed in vacuo and replaced with dichloromethane (30 mL) and the resulting suspension was filtered. Removal of the volatiles in vacuo once again gave **8** as a yellow solid (0.48 g, ca. 70%). Crystals suitable for X-ray diffraction were grown from dichloromethane. **Elemental analysis**: The recrystallised material did not give satisfactory elemental analysis, presumably because of the contamination of the material with inorganic salts; the data fitted **8**·1.45LiBr: calcd. (%) for C_32_H_26_AsP∙(LiBr)_1.45_: C 59.83, H 4.08; found: C 60.03, H 3.99. **^1^H NMR**: δ_H_ (300.1 MHz, CD_2_Cl_2_) 7.96 (2H, dd, ^3^*J*_HH_ = 7.2, ^3^*J*_HP_ = 4.5 Hz, H-8), 7.81–7.74 (2H, m, *o*-Ph), 7.59 (2H, d, ^3^*J*_HH_ = 7.3 Hz, H-2), 7.46–7.38 (2H, m, *m*-Ph), 7.38–7.31 (1H, m, *p*-Ph), 7.20 (4H, ^3^*J*_HH_ = 7.2 Hz, H-3 and H-7), 3.39–3.16 (8H, m, H-11 and H-12), 2.28 (2H, q, ^3^*J*_HH_ = 7.7 Hz, As-CH_2_), 1.54 (3H, t, ^3^*J*_HH_ = 7.7 Hz, CH_3_). **^13^C{^1^H} NMR** was not acquired due to low solubility of **8**. **^31^P{^1^H} NMR:** δ_P_ (121.5 MHz, CD_2_Cl_2_) δ_P_ = –26.5 (s). **IR** (KBr disc, cm^–1^): ν = 3071 w (ν_Ar–H_), 2962 s (ν_C–H_), 1325 s, 1260 vs, 1095 vs, br, 1022 vs, 802 vs, 735 s, 701 m. **Raman** (glass capillary, cm^–1^) ν = 3068 m (ν_Ar–H_), 2919 m (ν_C–H_), 1603 m, 1583 vs, 1565 s, 1330 vs, 997 s, 537 s, 253 m. **MS (CI+)**: *m/z* (%) 517.1 (58) [M + H], 487.1 (100) [M–Et]. **HRMS (CI+)**: *m/z* Calcd. for C_32_H_27_AsP (M + H): 517.1066, found 517.1059.

#### 4.2.6. (Ph_2_PAcenap)_2_AsEt (**9**)

TMEDA (0.51 mL, 3.4 mmol) was added to a suspension of **7** (750 mg, 1.32 mmol) in THF (35 mL) and the mixture was cooled to –78 °C. A solution of *n*-butyllithium (1.26 mL, 2.5 M in hexanes, 3.2 mmol) was added dropwise with stirring over 1 h. The solution was maintained at –78 °C for 30 min before being warmed up and maintained at 0 to 10 °C for an hour. The solution was re-cooled to –78 °C and a solution of chlorodiphenylphosphine (0.49 mL, 2.64 mmol) in THF (5 mL) was added dropwise over 45 min. The solution was left to warm to room temperature overnight with stirring. The volatiles were removed in vacuo and replaced with dichloromethane (30 mL), and the resulting mixture was filtered. Removal of the volatiles in vacuo once again gave **9** as a pale yellow solid (0.52 g, ~52%). Crystals suitable for X-ray diffraction were obtained in the form of monoxide **9a** from dichloromethane. **^1^H NMR**: δ_H_ (500.1 MHz, CD_2_Cl_2_) 7.51 (2H, d, ^3^*J*_HH_ = 7.0 Hz, H-2), 7.26–7.13 (12H, br m, H-7 and H-8 and *m*-Ph), 7.10–7.03 (2H, br m, H-3), 7.02–6.95 (4H, br m, *p*-Ph), 6.76 (8H, br s, *o*-Ph), 3.37–3.24 (8H, m, H-11 and H-12), 2.19 (2H, q, ^3^*J*_HH_ = 7.6 Hz, As-CH_2_), 1.32 (3H, t, ^3^*J*_HH_ = 7.6 Hz, CH_3_). **^13^C{^1^H} NMR**: δ_C_ NMR was not acquired due to low solubility of **9**. **^31^P{^1^H} NMR**: δ_P_ (121.5 MHz, CD_2_Cl_2_) δ_P_ = –19.9 (s). **IR** (KBr disc, cm^–1^): ν = 3044 m (ν_Ar–H_), 2962 vs, (ν_C–H_), 1601 s, 1477 s, 1261 vs, 1020 vs, br, 800 vs, 693 vs. **Raman** (glass capillary, cm^–1^) ν = 3047 vs, (ν_Ar–H_), 2930 s (ν_C–H_), 1604 s, 1583 s, 1325 vs, 998 vs, 536 vs. **MS (CI+)**: *m/z* (%) 779.1 (8) [M + H], 749.1 (16) [M–Et], 412.1 (32) [Ph_2_PAcenapAs]. **HRMS (CI+)** *m/z* Calcd. for C_50_H_42_P_2_As (M + H): 779.1978, found 779.1977.

## 5. Conclusions

Utilising *peri*-substitution, novel dative complexes bearing pnictogen dibromide acceptor groups have been isolated and fully characterised. While their stability resembles that of their chlorine analogues, compounds **3** and **4** are thermally stable up to well above 100 °C and can be stored as solids under an inert atmosphere indefinitely, which makes them interesting for further synthetic use. Using excess AsBr_3_ gave species **5**, in which a bromine atom of molecule **4** is ionically separated through interactions with co-crystallised molecules of AsBr_3_. The structures of **3**–**5** illustrate that in phosphine−pnictine complexes the local polarity (packing) effects within the crystal lattice result in rather different Pn−X bond distances due to the fine balance of the polar covalent and ionic bonding around the (formally) negatively charged pnictoranido motifs. This is further corroborated by computational evaluation of the close As∙∙∙As interaction observed in **4**, which is found to consist mostly of dispersion forces.

Isolation of compound **6** indicates disproportionationative redox instability of **5** at elevated temperatures, resulting in an unusual heterocubane dianion [As_6_Br_8_]^2–^, containing As(I) motifs.

Reaction of BrAcenapLi with EtAsI_2_ afforded no singly substituted species, only geminally disubstituted species **7**. This compound has been shown to be a useful synthon, with the As–C bonds being tolerant to the strong base *n*BuLi. Thus, treatment of **7** with *n*BuLi proceeds through lithium-halogen exchange; subsequent reactions with chlorophosphine electrophiles afforded the new species, cyclic **8** and acyclic **9**.

## Figures and Tables

**Table 1:** Selected bond lengths [Ångströms (Å)] and angles (degrees, °) for **3** to **9-O**.

**Compound**	**3** **·THF (E = P)**	**4** **·C_6_H_6_^[b]^ (E = As)**	**5 (E = As)**	**6 ^[c]^ (E = As)**
*peri*-region bond distances				
P9–E1	2.2701(16)	2.405(3) [2.407(3)]	2.3978(15)	2.365(4) [3.145(4)]
E1–Br1	2.6864(13)	2.6337(19) [2.6639(19)]	2.4375(9)	–
E1–Br2	2.4376(13)	2.6400(18) [2.634(2)]	3.000(9)	–
*peri*-region angles				
C9–P9–E1	97.92(14)	97.9(3) [98.4(3)]	97.78(18)	98.7(4)
C1–E1–P9	88.82(13)	84.9(3) [85.3(3)]	86.25(17)	85.7(3)
Br1–E1–Br2	172.22(5)	173.95(6) [172.27(6)]	175.3(2)	–
P9–E1–Br1	91.13(5)	96.01(9) [91.02(9)]	95.57(4)	–
P9–E1–Br2	96.38(5)	90.00(9) [95.95(9)]	88.77(4)	–
splay angle ^[a]^	–7.8(5)	–5(1) [–5(1)]	–4(1)	–5(1) [15(1)]
out-of-plane displacements				
P9	0.23	0.07 [0.10]	0.09	0.21 [0.12]
E1	–0.03	–0.36 [–0.26]	–0.01	–0.11 [–0.05]
dihedral angles				
P9–C9···C1–E	7.7(2)	10.9(4) [9.2(4)]	2.4(2)	2.8(5) [1.2(5)]
**Compound**	**7** **1/2CH_2_Cl_2_** **(E = Br)**	**8** **∙** **CH_2_Cl_2_ (E = P)**	**9-O (E = P) ^[f]^**	
*peri*-region bond distances				
As1–E9	3.23(1)	3.004(6)	3.176(5)	
As1–E29	3.28(1)	–	3.273(5)	
*peri*-region angles				
splay angle ^[a,d]^	17(1)	11.7(4)	16.1(8)	
splay angle ^[a,e]^	19(1)	11.4(4)	15.2(8)	
out-of-plane displacements				
As1	0.35, 0.02	0.19, 0.02	0.04, 0.71	
E9	–0.08	−0.03	−0.40	
E29	–0.01	−0.23	0.41	
dihedral angles				
As1–C1···C9–E9	12(1)	3.3(6)	8(1)	
As1–C21···C29–E29	1(1)	5.0(6)	27(1)	

[a] splay angle = sum of the bay region angles—360. [b] values in square parentheses are for the 2nd molecule in the asymmetric unit. [c] values in square parentheses refer to the P29 acenaphthene unit. [d] C10 acenaphthene unit. [e] C30 acenaphthene unit. [f] E29 in this structure represents P39.

## Data Availability

The research data underpinning this publication can be accessed at DOI https://doi.org/10.17630/7bae9495-1422-4bae-aa2b-d160ce8c1d3f.

## Supplementary Materials

The following are available online at, File S1: Experimental data, including general considerations, preparation of arsenic tribromide, X-ray diffraction and computational details [28,35,37,38,39,40,41,42,43,44,45,46,47,48,49,50,51,52,53,54,55,56,57]. Accession Codes CCDC 2116902-2116908 contain the supplementary crystallographic data for this paper. These data can be obtained free of charge via www.ccdc.cam.ac.uk/data_request/cif, accessed on 28 October 2021, or by emailing data_request@ccdc.cam.ac.uk, or by contacting The Cambridge Crystallographic Data Centre, 12 Union Road, Cambridge CB2 1EZ, UK; Fax: +44-1223-336033. The research data underpinning this publication can be accessed at DOI https://doi.org/10.17630/7bae9495-1422-4bae-aa2b-d160ce8c1d3f.

## Sample Availability

Samples of all compounds are not available from the authors.
