# Supplementary material for: Synthetic and Structural Study of peri-Substituted Phosphine-Arsines
Source: Molecules. 2021 Nov 28;26(23):7222. doi: 10.3390/molecules26237222 (PMC8658757; doi:10.3390/molecules26237222)
Supplement: Supplementary file 1 [file molecules-26-07222-s001.zip › molecules-1463594-supplementary/File S1.pdf]

Supplementary Material for: Synthetic and Structural Study of peri-Substituted Phosphine-Arsines

Brian A. Chalmers, D. M. Upulani K. Somisara, Brian A. Surgenor, Kasun S. Athukorala Arachchige, J. Derek Woollins, Alexandra M. Z. Slawin, Michael Bühl, Petr Kilian*

Further experimental details

All new compounds were characterised by ^{1}H, $^{13}C\{^{1}H\}$ and $^{31}P\{^{1}H\}$ NMR, including measurement of $^{1}H\{^{31}P\}$, H-H DQF COSY, H-C HSQC, H-C HMBC and H-P HMBC, unless specified otherwise. ^{13}C NMR spectra were recorded using the DEPT-Q-135 pulse sequence with broadband proton decoupling. Measurements were performed at 25 °C using a Bruker Avance III 500 (MHz) spectrometer or a Bruker Avance II 400 MHz spectrometer. 85% H_3PO_4 was used as an external standard for ^{31}P NMR; ^{1}H and ^{13}C NMR shifts are relative to Me_4Si, residual solvent peaks were used for calibration ($CHCl_3$ δ_H 7.26, δ_C 77.2 ppm, CD_2Cl_2 δ_H 5.32, δ_C 53.8 ppm, C_6H_6 δ_H 7.16, δ_C 128.1 ppm). Elemental analysis (C, H and N) was performed at London Metropolitan University. Mass Spectra were acquired by the EPSRC UK National Mass Spectrometry Facility (NMSF) at Swansea University, or by Mrs Caroline Horsburgh at the University of St Andrews. Infrared spectra were collected on a Perkin Elmer 2000 NIR FT spectrometer. "*In vacuo*" refers to a pressure of *ca.* 2×10^{-2} mbar.

Preparation of Arsenic Tribromide

Arsenic tribromide was prepared using a modification of the literature procedure.[1] Arsenic(III) oxide (20.0 g, 102 mmol) was dissolved in concentrated hydrobromic acid (160 mL) and heated to 120 °C with rapid stirring for *ca.* 20 minutes. The solution was cooled in ice and the aqueous (acid) layer decanted from the white solid that had collected. Hexane (100 mL) was added to the white solid to dissolve it. The aforementioned aqueous layer was washed with hexane (3 × 100 mL), and all the organic fractions were combined. The hexane solution was cooled in ice, and the solvent removed *in vacuo* with care as allowing the solution to get too warm results in loss of yield, as $AsBr_3$ sublimes readily. The isolated white solid was further purified by vacuum distillation using an air condenser (1.0 mbar, t_{oil} 85 °C, t_{vap} 50 °C) giving a white crystalline solid (50.2 g, 79%) (M. p. 31 °C). The solid

was stored in the fridge (0 °C) under nitrogen. **Raman data** (glass capillary, cm^{-1}) ν = 287m, 273vs (ν_{As-Br}), 263m. **MS** (EI): *m/z* (%) 313.68 (35) [M], 234.75 (100) [M–Br], 153.84 (5) [M–2Br].

X-ray Diffraction

Table S1 lists details of data collections and refinements. The crystallographic data for **3**, **6**, **7**, **8** and **9** were collected using a Rigaku MM007 Rotating Anode generator and Mercury70 diffractometer using monochromated Mo-Kα radiation at –180 °C (±1). The data for **4** were collected using a Rigaku SCX mini diffractometer using Mo-Kα radiation at –100 °C (±1). The data for **5** were collected using a Rigaku MM007 Rotating Anode generator and Saturn70 diffractometer using Mo-Kα radiation at –148 °C (±1). (Mo-Kα = λ = 0.71073 Å). Intensity data were collected using ω steps accumulating area detector frames spanning at least a hemisphere of reciprocal space. All data were corrected for Lorentz, polarisation and long-term intensity fluctuations. Absorption effects were corrected on the basis of multiple equivalent reflections. Non-hydrogen atoms were refined anisotropically and hydrogen atoms were refined using the riding model. The data for all compounds were collected and processed using *CrystalClear (Rigaku)*.[2, 3] The crystal structures were refined by full-matrix least-squares against F^2 (SHELXL)[4, 5] using the *CrystalStructure* GUI.[6] On-hydrogen atoms were refined anisotropically and hydrogen atoms were refined using the riding model. Searches of the *Cambridge Structure Database (CSD)* were performed using either *ConQuest* [7] or the *WebCSD*.[8] Images of crystal structures were obtained using *OLEX-2*[9] with all other manipulations carried out using *Mercury 3.8*.[10]

Table S1: Crystal and Structure Refinement Data.

	**3·THF**	**4·C$_6$H$_6$**	**5**	**6**
**Formula**	$C_{22}H_{30}Br_2OP_2$	$C_{24}H_{28}AsBr_2P$	$C_{18}H_{22}As_2Br_5P$	$C_{36}H_{44}As_4Br_4P_2$
Mr	532.23	582.19	818.71	1157.99
Colour/Habit	Colourless/prism	Colourless/prism	Yellow/chunk	Colourless/prism
**Crystal Dimensions [mm]**	0.100×0.050×0.030	0.214×0.202×0.201	0.150×0.120×0.020	0.030×0.030×0.030
**Crystal System**	Triclinic	Orthorhombic	Triclinic	Triclinic
**Space Group**	$P\bar{1}$	*Pbca*	$P\bar{1}$	$P\bar{1}$
***a* [Å]**	7.862(2)	13.199(9)	10.5185(11)	11.668(3)
***b* [Å]**	11.298(3)	21.452(14)	11.1333(10)	12.304(3)
***c* [Å]**	13.343(4)	33.37(2)	11.3740(11)	15.491(2)
**α [°]**	73.185(16)	90	76.955(11)	76.35(2)
**β [°]**	89.57(2)	90	79.891(12)	72.11(2)
**γ [°]**	81.51(2)	90	66.596(8)	80.92(2)
**V [Å^3]**	1121.3(5)	9450(11)	1185.3(2)	2047.8(8)
**Z**	2	16	2	2
**$\rho_{calcd.}$ [g cm^{-3}]**	1.576	1.637	2.294	1.878
**μ [cm^{-1}]**	37.78	49.08	113.43	72.54
**$2\theta_{max}$**	50.7	50.7	50.0	50.7
**F_{000}**	540.00	4640.00	772.00	1124.00
**Measured refln.**	7173	39482	8953	12939
**Unique refln.**	3970	8568	4104	7213
**R [I>2σ(I)]**	0.0463	0.0752	0.0425	0.0881
**wR**	0.0764	0.2111	0.1122	0.2258
**Goodness of Fit**	1.024	1.045	1.106	1.060
**Largest peak/hole [e Å^{-3}]**	0.69/–0.88	1.26/–0.80	0.98/–0.86	1.52/–1.32

	**7**·1/2CH$_2$Cl$_2$	**8**·CH$_2$Cl$_2$	**9-O**	
**Formula**	$C_{26.5}H_{22}AsBr_2Cl$	$C_{33}H_{28}AsCl_2P$	$C_{50}H_{41}AsO_{0.5}P_2$	
Mr	610.65	601.39	786.74	
Colour/Habit	Colourless/prism	Yellow/prism	Colourless/prism	
**Crystal Dimensions [mm]**	0.050×0.050×0.050	0.100×0.030×0.030	0.030×0.030×0.030	
**Crystal System**	Triclinic	Monoclinic	Monoclinic	
**Space Group**	$P\bar{1}$	$P2_1/c$	$P2_1/n$	
***a* [Å]**	7.887(3)	10.825(3)	12.240(4)	
***b* [Å]**	11.467(2)	13.859(3)	18.473(5)	
***c* [Å]**	13.733(4)	18.118(5)	16.745(5)	

**α [°]**	70.57(3)	90	90
**β [°]**	81.04(3)	106.990(7)	93.478(8)
**γ [°]**	81.93(3)	90	90
**V [Å^3]**	1151.7(7)	2599.6(11)	3779(2)
**Z**	2	4	4
**$\rho_{calcd.}$ [g cm^{-3}]**	1.761	1.536	1.383
**μ [cm^{-1}]**	50.84	15.98	10.22
**$2\theta_{max}$**	50.0	50.7	50.8
**F_{000}**	602.00	1232.00	1632.00
**Measured refln.**	7340	16475	23637
**Unique refln.**	4058	4690	6854
**R [I>2σ(I)]**	0.0688	0.0414	0.0666
**wR**	0.1878	0.0999	0.1322
**Goodness of Fit**	0.981	1.067	0.892
**Largest peak/hole [e Å^{-3}]**	1.89/–0.87	0.60/–0.89	0.46/–0.50

Computational Details

Geometries were fully optimized at the B3LYP-D3(BJ) level,[11-17] together with a fine integration grid (75 radial shells with 302 angular points per shell); As and Br were described with Curtis and Binning's 962(d) and 962+(d) bases,[18] respectively (a set of diffuse functions was used for the Br atoms because of the expected buildup of negative charge on them), 6-31G* basis was used elsewhere. This level is denoted B3LYP-D3/6-31(+)G*. Starting from the solid state structure, the dimer was first optimised at that "raw" level (without BSSE correction, affording a binding energy of ΔE = -19.3 kcal/mol), were computed harmonic frequencies were used to verify the nature of the minima and to obtain thermodynamic corrections to enthalpies and entropies at 298.15 K. The dimer was then reoptimised including a correction for basis-set superposition error (BSSE) according to the Counterpoise method,[19] using the two the monomeric molecular units as fragments. Using this optimised structure, WBIs[20] were obtained from a natural bond orbital (NBO) analysis[21] at the B3LYP/6-31(+)G* level. All computations were performed using the Gaussian09 suite of programs.[22]

Cartesian coordinates (xyz format in Å) of monomeric and dimeric 4, B3LYP-D3/6-31(+)G* optimised (including BSSE correction for the dimer).

44

monomer

Br	1.358633	2.923191	-0.618749
Br	0.475749	-1.156545	2.756635
As	1.026389	0.854268	1.036242
P	1.100161	-0.756314	-0.753100
C	-0.912394	0.911785	0.693895
C	-1.770349	1.776206	1.351651
H	-1.367646	2.503572	2.050623
C	-3.180095	1.743313	1.148687
H	-3.806592	2.444985	1.691930
C	-3.717801	0.818042	0.279517
C	-2.839538	-0.057659	-0.391632
C	-3.498470	-0.965841	-1.242417
C	-2.744407	-1.881004	-1.951530
H	-3.205094	-2.606869	-2.615125
C	-1.330837	-1.870287	-1.812641
H	-0.754393	-2.590819	-2.383802
C	-0.682050	-0.971347	-0.972160
C	-1.446232	-0.034045	-0.219541
C	-5.137419	0.496415	-0.153806
H	-5.614535	1.359644	-0.631547
H	-5.765484	0.224350	0.701757
C	-4.991146	-0.702143	-1.159438
H	-5.529006	-1.589269	-0.806115
H	-5.406920	-0.455691	-2.143040

C	1.801921	-0.042192	-2.319379
H	1.306727	0.930308	-2.399419
C	1.455158	-0.892196	-3.548251
H	1.877837	-1.902418	-3.490910
H	1.879534	-0.412173	-4.437135
H	0.375624	-0.972817	-3.695695
C	3.311585	0.200095	-2.187608
H	3.663758	0.726059	-3.081366
H	3.871224	-0.738335	-2.111972
H	3.545158	0.831258	-1.326130
C	2.044054	-2.336078	-0.441700
H	2.447151	-2.578383	-1.433697
C	1.160632	-3.498731	0.027081
H	0.691609	-3.270145	0.985792
H	1.792629	-4.385761	0.150443
H	0.377040	-3.738674	-0.695714
C	3.224088	-2.084800	0.513456
H	3.843229	-2.987870	0.555579
H	2.861207	-1.868874	1.520794
H	3.860153	-1.258047	0.184136

88

dimer

Br	1.012138	-1.934911	-1.753059
Br	2.140293	1.598480	2.154147
As	1.632547	-0.133189	0.131161
P	3.135955	1.069295	-1.328167
C	3.225205	-1.147924	0.671571
C	3.163238	-2.222279	1.543583
H	2.195040	-2.561868	1.899796
C	4.337150	-2.891738	1.994783
H	4.234653	-3.730051	2.678090
C	5.573946	-2.456941	1.566317
C	5.629355	-1.364289	0.676408
C	6.941243	-0.999214	0.318483
C	7.123975	0.063670	-0.545145
H	8.115531	0.389393	-0.845932
C	5.986948	0.744844	-1.057278
H	6.149722	1.569882	-1.743568
C	4.691256	0.382502	-0.703607
C	4.487331	-0.697919	0.203551
C	6.994919	-2.911996	1.851354
H	7.151323	-3.951047	1.539612
H	7.223087	-2.868253	2.922165
C	7.906943	-1.928755	1.031778
H	8.581525	-1.367870	1.688828
H	8.541590	-2.467601	0.318970
C	2.946553	0.586551	-3.113615
H	2.875691	-0.504537	-3.077101
C	4.168157	0.989790	-3.949138

H	4.320905	2.075739	-3.966402
H	4.005198	0.669125	-4.984232
H	5.084134	0.513747	-3.590787
C	1.636793	1.135144	-3.696328
H	1.496561	0.716202	-4.698642
H	1.660976	2.225712	-3.794058
H	0.762882	0.855163	-3.103604
C	3.036105	2.931112	-1.288381
H	3.301481	3.214637	-2.315123
C	4.035883	3.576503	-0.321257
H	3.836741	3.268219	0.707293
H	3.928288	4.665665	-0.384372
H	5.070500	3.322745	-0.565872
C	1.590957	3.384561	-1.014919
H	1.521043	4.465155	-1.184252
H	1.324831	3.182168	0.024283
H	0.857888	2.896037	-1.662291
Br	-2.138782	1.566815	-2.177264
Br	-1.012192	-1.904472	1.785377
As	-1.632009	-0.133652	-0.127677
P	-3.137892	1.089331	1.311787
C	-3.223029	-1.157886	-0.654635
C	-3.159213	-2.244454	-1.511195
H	-2.190340	-2.587972	-1.861759
C	-4.332080	-2.921566	-1.953659
H	-4.228151	-3.769313	-2.625006
C	-5.569668	-2.482164	-1.532236
C	-5.626908	-1.377093	-0.657901
C	-6.939423	-1.008494	-0.305983
C	-7.123968	0.066432	0.542232
H	-8.116085	0.395259	0.837749
C	-5.988085	0.756346	1.045060
H	-6.152196	1.591098	1.719175
C	-4.691734	0.390451	0.697467
C	-4.485963	-0.702709	-0.193877
C	-6.989994	-2.942418	-1.812193
H	-7.145707	-3.977513	-1.487253
H	-7.217352	-2.912666	-2.883666
C	-7.903595	-1.949429	-1.006186
H	-8.579436	-1.398988	-1.670708
H	-8.536966	-2.479561	-0.285739
C	-3.041519	2.950471	1.242855
H	-3.308811	3.249286	2.264743
C	-4.041429	3.578541	0.264554
H	-3.841501	3.253397	-0.758648
H	-3.935182	4.668729	0.309550
H	-5.075854	3.327615	0.512886
C	-1.596814	3.402485	0.964538
H	-1.529070	4.485545	1.118265
H	-1.328484	3.185460	-0.071142
H	-0.863949	2.924655	1.620041

C	-2.948315	0.636184	3.105108
H	-2.873683	-0.455104	3.086775
C	-4.172391	1.049278	3.932270
H	-4.330642	2.134574	3.928375
H	-4.008537	0.749433	4.973443
H	-5.085763	0.561897	3.582630
C	-1.641144	1.198516	3.680797
H	-1.500637	0.795061	4.689392
H	-1.669734	2.290316	3.762327
H	-0.765274	0.913209	3.093438

References

1. Arnaiz, F. J.; Miranda, M. J.; Rheingold, A. L., Arsenic (III) Bromide. *Inorg. Synth.* **2002,** *33*, 203.
2. CrystalClear 2.0, Rigaku Corporation, The Woodlands, TX, USA. 2007.
3. CrystalClear Software User's Guide, Molecular Structure Corportation ©. 2007.
4. Sheldrick, G., Crystal structure refinement with SHELXL. *Acta Crystallographica Section C* **2015,** *71* (1), 3-8.
5. Sheldrick, G., SHELXT - Integrated space-group and crystal-structure determination. *Acta Crystallographica Section A* **2015,** *71* (1), 3-8.
6. CrystalStructure 4.3.0; Rigaku Americas: The Woodlands, TX, USA, Rigaku Corporation: Tokyo, Japan, **2018**.
7. Bruno, I. J.; Cole, J. C.; Edgington, P. R.; Kessler, M.; Macrae, C. F.; McCabe, P.; Pearson, J.; Taylor, R., New software for searching the Cambridge Structural Database and visualizing crystal structures. *Acta Crystallographica Section B* **2002,** *58* (3 Part 1), 389-397.
8. Thomas, I. R.; Bruno, I. J.; Cole, J. C.; Macrae, C. F.; Pidcock, E.; Wood, P. A., WebCSD: the online portal to the Cambridge Structural Database. *Journal of Applied Crystallography* **2010,** *43* (2), 362-366.
9. Dolomanov, O. V.; Bourhis, L. J.; Gildea, R. J.; Howard, J. A. K.; Puschmann, H., OLEX2: a complete structure solution, refinement and analysis program. *Journal of Applied Crystallography* **2009,** *42* (2), 339-341.
10. Macrae, C. F.; Bruno, I. J.; Chisholm, J. A.; Edgington, P. R.; McCabe, P.; Pidcock, E.; Rodriguez-Monge, L.; Taylor, R.; van de Streek, J.; Wood, P. A., Mercury CSD 2.0– new features for the visualization and investigation of crystal structures. *Journal of Applied Crystallography* **2008,** *41* (2), 466-470.
11. Becke, A. D., Density‐functional thermochemistry. III. The role of exact exchange. *The Journal of Chemical Physics* **1993,** *98* (7), 5648-5652.
12. Lee, C.; Yang, W.; Parr, R. G., Development of the Colle-Salvetti correlation-energy formula into a functional of the electron density. *Physical Review B* **1988,** *37* (2), 785-789.
13. Grimme, S.; Antony, J.; Ehrlich, S.; Krieg, H., A consistent and accurate ab initio parametrization of density functional dispersion correction (DFT-D) for the 94 elements H-Pu. *The Journal of Chemical Physics* **2010,** *132* (15), 154104.
14. Grimme, S.; Ehrlich, S.; Goerigk, L., Effect of the damping function in dispersion corrected density functional theory. *Journal of Computational Chemistry* **2011,** *32* (7), 1456-1465.
15. Risthaus, T.; Grimme, S., Benchmarking of London Dispersion-Accounting Density Functional Theory Methods on Very Large Molecular Complexes. *Journal of Chemical Theory and Computation* **2013,** *9* (3), 1580-1591.
16. Becke, A. D.; Johnson, E. R., Exchange-hole dipole moment and the dispersion interaction. *The Journal of Chemical Physics* **2005,** *122* (15), 154104.

17. Johnson, E. R.; Becke, A. D., A post-Hartree-Fock model of intermolecular interactions: Inclusion of higher-order corrections. *The Journal of Chemical Physics* **2006,** *124* (17), 174104.
18. Binning, R. C.; Curtiss, L. A., Compact contracted basis sets for third-row atoms: Ga–Kr. *Journal of Computational Chemistry* **1990,** *11* (10), 1206-1216.
19. Boys, S. F.; Bernardi, F., The calculation of small molecular interactions by the differences of separate total energies. Some procedures with reduced errors. *Molecular Physics* **1970,** *19* (4), 553-566.
20. The WBI is a measure for the covalent character of a bond and adopts values close to 1 and 2 for true single and double bonds, respectively: Wiberg, K. B., Application of the pople-santry-segal CNDO method to the cyclopropylcarbinyl and cyclobutyl cation and to bicyclobutane. *Tetrahedron* **1968,** *24* (3), 1083-1096.
21. Reed, A. E.; Curtiss, L. A.; Weinhold, F., Intermolecular interactions from a natural bond orbital, donor-acceptor viewpoint. *Chemical Reviews* **1988,** *88* (6), 899-926.
22. Frisch, M. J.; Trucks, G. W.; Schlegel, H. B.; Scuseria, G. E.; Robb, M. A.; Cheeseman, J. R.; Scalmani, G.; Barone, V.; Mennucci, B.; Petersson, G. A.; Nakatsuji, H.; Caricato, M.; Li, X.; Hratchian, H. P.; Izmaylov, A. F.; Bloino, J.; Zheng, G.; Sonnenberg, J. L.; Hada, M.; Ehara, M.; Toyota, K.; Fukuda, R.; Hasegawa, J.; Ishida, M.; Nakajima, T.; Honda, Y.; Kitao, O.; Nakai, H.; Vreven, T.; Montgomery Jr., J. A.; Peralta, J. E.; Ogliaro, F.; Bearpark, M. J.; Heyd, J.; Brothers, E. N.; Kudin, K. N.; Staroverov, V. N.; Kobayashi, R.; Normand, J.; Raghavachari, K.; Rendell, A. P.; Burant, J. C.; Iyengar, S. S.; Tomasi, J.; Cossi, M.; Rega, N.; Millam, N. J.; Klene, M.; Knox, J. E.; Cross, J. B.; Bakken, V.; Adamo, C.; Jaramillo, J.; Gomperts, R.; Stratmann, R. E.; Yazyev, O.; Austin, A. J.; Cammi, R.; Pomelli, C.; Ochterski, J. W.; Martin, R. L.; Morokuma, K.; Zakrzewski, V. G.; Voth, G. A.; Salvador, P.; Dannenberg, J. J.; Dapprich, S.; Daniels, A. D.; Farkas, Ö.; Foresman, J. B.; Ortiz, J. V.; Cioslowski, J.; Fox, D. J. *Gaussian 09*, Gaussian, Inc.: Wallingford, CT, USA, 2009.
